# Transcultural adaptation and psychometric validation of a Spanish-language version of the “Pelvic Girdle Questionnaire”

**DOI:** 10.1186/s12955-017-0605-0

**Published:** 2017-02-01

**Authors:** Montserrat Rejano-Campo, Raúl Ferrer-Peña, M. Alicia Urraca-Gesto, Tomás Gallego-Izquierdo, Daniel Pecos-Martín, Britt Stuge, Gustavo Plaza-Manzano

**Affiliations:** 1 Physiotherapy and Nursery Department, Alcalá University, Madrid, Spain; 2 Department of Physiotherapy, Faculty of Health Science, The Center for Advanced Studies University La Salle, Universidad Autónoma de Madrid, Madrid, Spain; 3 Department of Rehabilitation and Physiotherapy, University Hospital Alcorcon Foundation, Madrid, Spain; 4 Department of Orthopaedics, Oslo University Hospital, Oslo, Norway; 5 Departamento de Medicina Física y Rehabilitación, Facultad de Medicina, Universidad Complutense de Madrid, Ciudad Universitaria, Madrid, 28040 Spain; 6 Instituto de Investigación Sanitaria del Hospital Clínico San Carlos (IdISSC), Madrid, Spain

**Keywords:** Pelvic girdle pain, Pelvic Girdle Questionnaire, Validation studies, Cultural adaptation, Postpartum

## Abstract

**Background:**

The Pelvic Girdle Questionnaire is the only instrument designed to assess pain and disability specifically in pregnant or postpartum women with pelvic girdle pain. The objective of this study was the adaptation to the Spanish language and analysis of the psychometric properties of the Pelvic Girdle Questionnaire.

**Methods:**

This is a descriptive cross-sectional study divided into two phases. In the first phase, a translation and adaptation process was performed according to international guidelines. Secondly, the analysis of the properties of the Spanish version was conducted using a sample of 125 pregnant or postpartum women suffering from pelvic girdle pain. Participants completed the Spanish version along with five other measurement instruments through an online platform. Internal consistency, construct validity, test-retest reliability, the ceiling and floor effects, responsiveness and discriminatory ability of the Spanish version were analysed.

**Results:**

The Spanish version of the *Pelvic Girdle Questionnaire* showed high internal consistency with Cronbach's alpha = 0.961, and an intraclass correlation coefficient of 0.962. The convergent validity showed high positive correlation with other questionnaires used. ROC curves showed no discriminatory capacity for number of sites of pain or pregnancy/post-partum state.

**Conclusions:**

This article presents the translation, validation and psychometric properties of the Spanish version of the *Pelvic Girdle Questionnaire,* that has proved to be an appropriate and valid assessment tool of disability due to pelvic girdle pain in pregnant and postpartum women.

**Electronic supplementary material:**

The online version of this article (doi:10.1186/s12955-017-0605-0) contains supplementary material, which is available to authorized users.

## Background

Pelvic girdle pain (PGP) is characterized by the presence of pain in the posterior region of the pelvis, distal and lateral to the fifth lumbar vertebra, and/or at the level of the pubic symphysis [1]. It occurs mainly in women during pregnancy or in the postpartum period. PGP is provoked or increased by everyday activities such as walking, standing, sitting and laying down, and can be increased after only 30 min of activity, limiting almost daily and working abilities [2]. 20% of women report pregnancy-related PG [3], but incidence of pain in the pelvic girdle in pregnancy varies from 4–76% depending principally on the definition and the diagnostic tools [4] The PGP sick-leave in pregnancy ranges from 37–72%, and length of sick leave is 12–15 weeks average [4]. Most of these women recover quickly after giving birth, but between 5 and 7% continue with these symptoms in more advanced postpartum stages.

Vleeming et al. [5] insist on the importance of differentiating between pelvic girdle pathology and lumbar pathology due to a prognosis and a management that seem different [6–8] To our knowledge, the Pelvic Girdle Questionnaire (PGQ) is the only questionnaire to assess pain and disability specifically in patients with PGP [9]. There is a need of a tool able, on one side, to optimize specific diagnosis and management in PGP and, on the other side, to give clinical investigation a tool to evaluate the actual treatments used to manage PGP. There is no scale or questionnaire validated to Spanish that fits these requisites.

The PGQ consists of 25 items: 20 items measuring activity limitations and 5 for measuring symptoms. Each item can be scored using a Likert-type scale of 4 points. The theoretical range is 100 points, 100 representing the most serious condition. So far the PGQ has been validated in English and Norwegian [10].

The main objective of this study is the transcultural adaptation and validation of the Spanish-language version of the PGQ in a sample of pregnant and postpartum period patients and the analysis of the psychometric properties of the Spanish version.

## Methods

### Design

Adaptation and validation of the PGQ was conducted through a descriptive cross-sectional study. This was carried out in two phases: an initial phase in which the translation and cultural adaptation of the questionnaire was conducted and a second validation phase in which the behaviour of the psychometric characteristics in 125 participants was conducted.

All research procedures used in this study were established in accordance with the Declaration of Helsinki and were approved by the Ethics Committee of the University of Alcalá de Henares (CEI: CEIM / HU / 2014/015).

### Participants

Selection guidelines were given to a group of 19 experienced specialists in Urogynaecology and / or Physical Therapy working in private practice. These guidelines include: eligibility criteria, guidelines for clinical tests and instructions for the implementation of the questionnaire. Women suffering from PGP attended the consultation of one of the 19 specialists collaborating in our study. A total of 125 women between 18 and 50 years of age were selected to participate in the study according to the following inclusion and exclusion criteria:

Inclusion criteria: women between 18 and 50 years-old, pregnant or having given birth less than one year earlier, with PGP whose onset occurred during pregnancy or within 3 weeks after birth.

Exclusion criteria: radicular pain below the knee, hip disease, previous surgery on the spine, pelvis or lower limbs, chronic vaginismus-type pelvic pain, spondylolisthesis or severe lumbar disease, inflammatory diseases, prolapse or severe urinary disease, impossibility or difficulty in understanding the questionnaires and suspected serious pathology (weakness of the lower limbs, reflex changes or loss of sensation associated with the same spinal nerve).

**Fig. 1:**
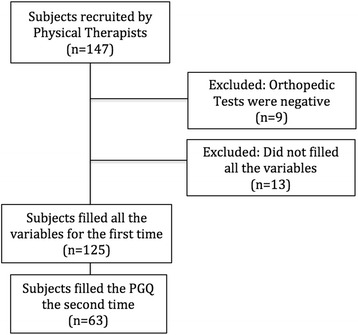
Recruitment process

Figure 1 represents the recruitment process.

Specialists who evaluated the patients used as reference the following battery of tests for diagnosis of PGP:

1. Posterior pelvic pain provocation test: the subject lies supine with a 90° hip flexion. The examiner exerts slight pressure on the femur while stabilizing the subject’s pelvis. The test is positive if the patient experiences a similar pain to that usually suffered. Evaluation of this test showed a specificity of 80% and a sensitivity of 81% [11].
2. Active straight leg raise test: The patient is placed supine with legs extended and feet 20 centimetres apart. The patient must lift one leg off the table up to 20 centimetres high without bending the ankle. The patient must express from 0 to 5 the difficulty experienced, with 0 being the minimum difficulty and 5 being impossible. This test has been validated by Mens et al. [12]. Its sensitivity is 87% and its specificity is 91%.
3. Long dorsal sacroiliac ligament provocation test: a bilateral palpation is performed at the level of the long dorsal sacroiliac ligament, which is slightly below the posterior superior iliac spine. The patient must express the pain experienced on a scale of 0 to 3. The sum of the scores obtained on each side gives a value from 0 to 6. This test has been validated by Vleeming et al. [13]. Its sensitivity in women with PGP is 76%, and 98% when the subject has severe pain.
4. Provocation of pain in the pubic symphysis by palpation: With the subject positioned supine, the examiner should perform a direct palpation over the pubic symphysis. If palpation causes pain for more than 5 s after removal of the stimulus, the test is positive. The sensitivity of this test is 81% and the specificity 99% [14].
5. Modified Trendelenburg test: the subject stands on one leg and, with the raised leg, performs a 90° flexion of the hip and knee. If the patient experiences pain at the pubic symphysis, the test is positive. This test has a sensitivity of 60% and a specificity of 97% [15].
6. Participants were included in the study provided the first two tests and at least one of the other three were positive.

All study participants signed an informed consent and were explained, through an information sheet, the implications of participating in the study.

### Translation and transcultural adaptation

**Fig. 2:**
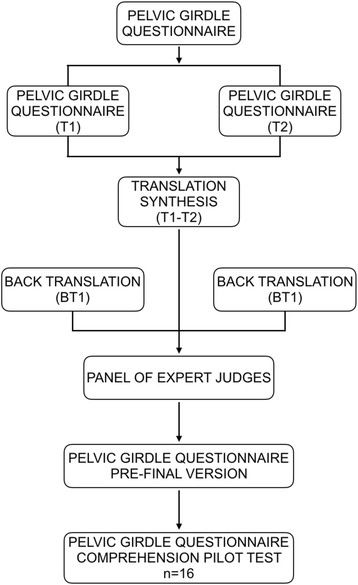
Flowchart of the process of translation and adaptation of the PGQ

The transcultural adaptation of the questionnaire was performed following the structured procedure of 5 phases described by Beaton et al. [16] (Fig. 2). In the first stage the synthesis of two translations of the questionnaire from English into Spanish was made. 2 experts in linguistics, one of them was a clinician, and 2 native English speakers with a complete management of Spanish language performed the firsts phases of translations. Content validity was performed on the prefinal version of the questionnaire by a panel of 6 expert judges, including specialists in psychometry, linguistics and chronic pain. Criteria for the selection of the experts were [17]: a) experience on the realization of judgments and decision making based on evidence (investigations, publications and experience); b) availability and motivation to participate, and c) impartiality and adaptability. Members of the panel of experts were asked to conduct a qualitative evaluation of every item (degree of understanding, agreement with the text), and were also asked to conduct a quantitative assessment of each item following theses criteria: 1) competence (items belong to theoretical established factors); 2) clarity (item is easily understood, its semantics and syntactics are suitable); 3) coherence (item has a relation with the factor being measured), and 4) relevance (item is essential and has to be included). Every point to assess about each item was quantified by a Likert- type scale of 3 points: (1: Agree; 2: Neither agree nor disagree; 3: Disagree). Once established and analyzed the extracted information from the judges’ panel we proceed to make a pilot comprehension test of the instrument with a sample of 16 patients. Patients evaluated each and every 25 elements using a two-values scale (I understand/ I don´t understand) to express any understanding problem. The pilot comprehension test performed was satisfactory, 11 patients (68,75%) rated all items as comprehensive and item 23 (“leg/s giving way”) was the least understood rated negative by 3 patients (18,75%). Complete results of the pilot comprehension test are presented in Table 1.

**Table 1:** Descriptive results of the pilot comprehension test (*N* = 16)

Patient Number	Items Understood N(%)	Items Not Understood
Patient 1	24 (96%)	item 23
Patient 2	25 (100%)	-
Patient 3	25 (100%)	-
Patient 4	25 (100%)	-
Patient 5	25 (100%)	-
Patient 6	25 (100%)	-
Patient 7	24 (96%)	item 23
Patient 8	25 (100%)	-
Patient 9	25 (100%)	-
Patient 10	24 (96%)	item 23
Patient 11	25 (100%)	-
Patient 12	25 (100%)	-
Patient 13	25 (100%)	-
Patient 14	23 (92%)	item 2 & 16
Patient 15	23 (92%)	item 19 & 20
Patient 16	25 (100%)	-

### Validation of the Spanish version

125 patients participated in the study who were selected between January and August 2015. Participants were provided a link to the online data collection platform *SurveyMonkey*, which gave them access to the questionnaires. This platform follows the security protocols defined by European agreements on the protection of personal data. This procedure was performed following the guidelines recommended by Eysenbach et al. [18].

The participants accessed the questionnaire by entering a 5-digit code provided by the specialist in charge. This code was unique to each professional and allowed us to identify each participant’s referent. In addition, they were asked 3 initial verification questions: age, sex and location of pain. These questions were intended to ensure the representativeness of the participants by checking that the subject corresponded to the established participation criteria. In order to avoid the same person filling out the questionnaire more than once, access was controlled based on the IP (internet protocol) address.

The participants completed a questionnaire on sociodemographic data, the Spanish version of the PGQ (Additional file 1) and a battery of self-report instruments comprising: the Oswestry disability index [19] for lumbar pain, the Spanish version of the “Fear Avoidance Beliefs” questionnaire [20], the pain catastrophizing scale [21], 8-item version of the SF-36 (SF 8) in Spanish [22] and a numerical scale for pain assessment [23].

### Data analysis

Calculation of the sample size was performed according to the criteria established by Kline et al. [24], which recommend a ratio of 5–10 subjects per item.

The data analysis was performed using version 22.0 of the SPSS statistical software (SPSS Inc. Chicago, IL, USA). Descriptive statistics of the quantitative variables are presented as the mean, standard deviation, range and confidence interval at 95%. The descriptive analysis on the scores obtained in each questionnaire are presented with the median and the first, third quartile, as they are ordinal variables.

The statistical analysis to test the psychometric properties was performed by:

- *Content validity*. It is calculated using the Aiken´s V index [25]. For a valid item a coefficient of 0.7 or more [26] must be achieved.
- *Internal consistency.* It was measured by Cronbach's alpha. A value below 0.7 indicates low consistency, 0.7 to 0.8 moderate and more than 0.8 indicates good internal consistency [27].
- *Ceiling and floor effects.* For analysis, the percentage of patients with the lower and higher score in each dimension are taken into account. It is considered that the ceiling and floor effects are present if more than 15% of respondents achieved the highest or lowest total score possible [28].
- *Convergent validity*. It was assessed by the Pearson correlation coefficient. The correlations were measured with the following instruments: the Oswestry disability index for low back pain, the "Fear Avoidance Beliefs" questionnaire, the pain catastrophizing scale, the SF 8 scale and a numerical scale.
- *Factor Analysis*. The Kaiser-Meyer-Olkin test and the Bartlett sphericity test were performed to determine if the sample was suitable for carrying out the exploratory factor analysis. Subsequently the factorial structure was analysed by exploratory factor analysis (first without rotating and then a rotated and forced solution with two components on theoretical grounds). On the number of factors to be retained, a minimum limit of eigenvalues ≥ 1. Those items that sufficiently saturated the retained factors were selected, setting a limit of 0.30 or higher for the selection. The analysis was completed with a scree plot.
- *Discriminatory ability*. It was calculated using the ROC curve (Receiver Operating Characteristic). The discriminatory power of the instrument was assessed in respect of 1) pregnant women / women in the postpartum period and 2) location of pain in 1 or 2 areas / location of pain in 3 areas. The range of the ROC curve scores from 0.50 (representing no discriminatory power whatsoever) to 1.0 (perfect discriminatory power) [29].
- *Test-retest reliability*. The sensitivity was analysed by the intraclass correlation coefficient (ICC). For this calculation, subjects completed the questionnaire twice with a 7–10-day interval of time between each one. A ICC value above 0.80 is considered aceptable [28].
- *Standard Error of Measurement (SEM).* It is calculated using the formula SEM = DT√ (1-ICC) where DT is the standard deviation and ICC intraclass correlation coefficient [30, 31].

## Results

The Spanish version of the PGQ was finally fulfilled by 125 women selected to participate in the study, 85 women were pregnant (65.4%) and 43 were postpartum women (34.4%). It presented the following characteristics: mean age 31.26 (SD 4.83) years (range 20–42); weight 69.29 (SD 12.93) kg. (range 45–117); Height 163.58 (SD 6.74) cm (range 145–182); BMI 24.67 (SD 6.24) kg.m-2; EVA 3.94 (SD 2.31); areas of pain: 72% pain in one area (36% at the level of the pubis, 14.4% left posterior area, 22.4% right posterior area), 23.2% pain in two areas (4% pubis + posterior left, 5.6% pubis + posterior right and 12.8% posterior left and right) and 4.8% pain in three area Table 2. Descriptive data of the 25 items are in Table 3.

**Table 2:** Descriptive Data of the sample (*n* = 125)

Variable	Frequency	Variable	Frequency
Pregnant woman	82 (65.5%)	Postpartum woman	43 (34.4%)
^b^Pain areas 1/2/3	90 (72.00%), 29 (23.2%),6 (4.8%)	^a^Civil state M/UM	38,87 (30.40),87 (69.60)
	Mean (SD)		Mean (SD)
Age	31,26 (4.83)	Weight	69,29 (12,93)
Height	163,58 (6.74)	BMI	24,67 (6.24)
VAS	3,94 (2.31)		

^a^Civil state: M: married UM: unmarried

^b^Pain areas: 1 = one pain area, 2 = two pain areas, 3 = three pain areas

**Table 3:** Descriptive Data of the The Pelvic Girdle Questionnaire items (*n* = 125)

Ítem*	score	Ítem*	score
1	0.82 (0.824)	2	0.84 (0.846)
3	1.73 (0.995)	4	1.44 (0.911)
5	0.54 (0.724)	6	1.26 (1.025)
7	0.75 (0.779)	8	1.65 (0.969)
9	1.30 (0.969)	10	1.29 (0.811)
11	0.74 (0.742)	12	1.70 (0.916)
13	1.58 (0.960)	14	1.01 (0.955)
15	1.80 (1.055)	16	1.70 (1.063)
17	1.12 (0.997)	18	1.50 (1.044)
19	1.20 (1.032)	20	1.05 (0.949)
21	1.19 (0.748)	22	1.86 (0.786)
23	0.87 (0.889)	24	1.40 (0.907)
25	1.32 (1.021)		

* Values are mean and standard deviation

### Content validity

Regarding content validity calculated by the Aiken´s V index, the 25 items assessed by experts presented values greater than 0.70, so none were rejected. The items number 14, 18 and 23 had the lowest values, with a score of 0.75. Items 4,8,13,15,16,21 and 22 obtained a score of 1, this being the maximum value.

The median duration of response of the Spanish version of the PGQ was 03:00, with a standard deviation of 01:42, a lower range of 01:00 and a higher range of 08:31. To perform this calculation outliers over 1 h were excluded as not being deemed valid.

### Internal consistency

Total Cronbach's alpha coefficient for the questionnaire was 0.961. Cronback´s alpha coefficient for items referred to activity (1–20) is 0.961 and 0.960 for questions dedicated to symptoms (21–25).

### Floor and ceiling effect

The Spanish version of the questionnaire did not present a ceiling or floor effect, since no subject scored the minimum (0 points) or the maximum (100 points).

### Convergent validity

The Pearson correlation coefficient showed a high positive correlation of the Spanish-language version of the PGQ with the rest of the questionnaires (Table 4).

**Table 4:** Pearson correlations between instruments

	PGQ_TOTAL	PGQ_SubAct	PGQ_SubSint	*P*
PGQ_TOTAL	1	.994^**^	.917^**^	<0.05
PGQ_SubAct	.994^**^	1	.870^**^	<0.05
PGQ_SubSint	.917^**^	.875^**^	1	<0.05
ODI_TOTAL	.838^**^	.814^**^	.845^**^	<0.05
FAB_TOTAL	.530^**^	.511^**^	.549^**^	<0.05
PCS_TOTAL	.768^**^	.748^**^	.771^**^	<0.05
PCS_Rum	.729^**^	.711^**^	.722^**^	<0.05
PCS_Mag	.601^**^	.589^**^	.587^**^	<0.05
PCS_Help	.777^**^	.752^**^	.794^**^	<0.05
VAS	.762^**^	.750^**^	.733^**^	<0.05
SF8	.805^**^	.792^**^	.775^**^	<0.05

PGQ SubAct: activity subscale of the PGQ; PGQ SubSint: symptom subscale of the PGQ; ODI: Oswestry Disability Index, FAB: « Fear Avoidance Beliefs » Questionnaire, PCS: pain catastrophizing scale, PCS_Rum: Pain Catastrophizing Subscale Rumination PCS_Mag: Pain Catastrophizing Subscale Magnification PCS_Help: Pain Catastrophizing Subscale Helplessness VAS: Visual Analog Scale. SF 8 : 8-items version of the SF-36

**Correlation significance at *p*<0.01 (two tails)

### Factor analysis

A value of 0.903 was obtained in the Kaiser-Meyer-Olkin and Bartlett sphericity test (*p* < 0.001), so it was determined that the sample was suitable for carrying out the exploratory factor analysis.

**Fig. 3:**
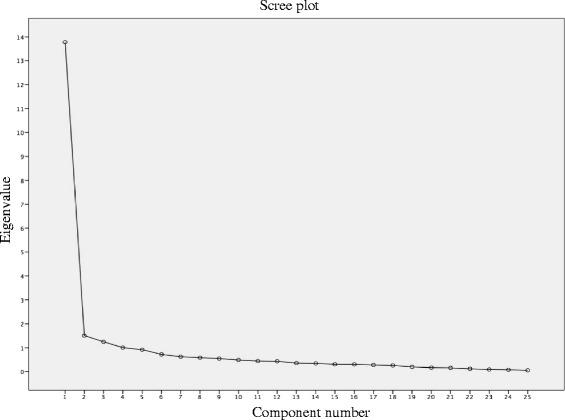
Scree plot

Initially an exploratory factor analysis was conducted using principal components without rotation. Additionally, a forced solution with two components was rotated using the Varimax method. The results showed a percentage of explained variance of 55.08% for a single factor, while a second factor explained a variance of 6.03%. The cumulative percentage of two components was 61%. We opted for a one-dimensional solution based on the values of the principal components matrix (Table 5) and observing the scree plot (Fig. 3).

**Table 5:** Forced solution with 2 components and Rotated Total explained variance

	Extraction Sums of Squared Loading			Rotation Sums of Squared Loading
		%	%	
Component	Total	variance	accumulated	Total
1	13.772	55.086	55.086	12.320
2	1.509	6.037	61.123	11.396

### Discriminatory capacity

- Pregnant / Postpartum women. It was calculated by taking the group of pregnant women as the positive real state. The AUC was 0.51, with a standard error of 0.05. Therefore, it was considered that the Spanish version of the PGQ is unable to differentiate between the two groups.
- 3 areas of pain / 1 or 2 areas of pain. It is calculated by taking "three areas of pain" as the positive real state. Although the values of the graph showed a proportional tendency between area of pain and amount of pain, only 6 patients reported pain in the 3 areas at the same time, the rest of the sample had pain in 1 or 2 areas of the pelvis. Therefore not sufficient significance was found to confer a discriminatory capacity by area of pain to our questionnaire.

### Test-retest reliability

It was calculated with a random sub-sample of 62 patients who completed the Spanish version of the PGQ between 7 and 10 days later. The reliability of the instrument assessed by the ICC showed very high levels of consistency (ICC = 0.962), representing a satisfactory temporal stability.

### Standard error of measurement

The SEM was 3.23% error. This percentage represents the discrepancy between the results of a particular evaluation and the average of all the results that a person could hypothetically get.

## Discussion

In the present study the transcultural adaptation and validation of the Spanish-language version of the PGQ is performed. Statistical analysis of this validation showed a high level of internal consistency and high reliability of the instrument in the test-retest.

The process of adaptation of this questionnaire allowed us to obtain a semantically and conceptually similar version to the original. Like the PGQ, our version consists of 25 items and is divided into two sections: the first consists of 20 items measuring the activity limitations, and the second contains 5 items measuring symptoms. Each item can be scored using a Likert-type scale of 4 points. The range is from 0 to 100, with 100 being the most serious condition.

In terms of methodology, the specialists responsible for the selection of the sample used reference tests to determine the inclusion of the participants. This procedure was similar to that used in the study of Stuge et al. [9]. With regard to the questionnaire support, Stuge et al. [9] and Grotle et al. [10] used a postal system, while our study used an online platform in accordance with the *Checklist for Reporting Results of Internet E-Surveys* [18]. Regarding the statistical analysis, it is important to note that the sample size was different in the two studies. While Grotle’s study involved 87 subjects (42 performed the retest), our sample had 125 participants (62 performed the retest).

As regards content validity, the 25 items presented values greater than 0.70. In reason of these content-relevance ratings, all of the items were considered necessary and none were rejected [25].

The internal consistency of the Spanish version has been higher than that of the original tool. The original questionnaire presented a Cronbach's alpha of 0.86, while the Castilian version has reached a value of 0.96. This coefficient, greater than 0.9, indicates a suitable value of consistency [32], but it may also suggest a degree of redundancy among the items. A high association after removing each item reflects redundancy since the components of each dimension aim to reflect independent values. Despite this high figure, it was decided to keep all the items mainly for two reasons: first, the values of the content validity were adequate and all items were considered necessary and, second, keeping all the items ensures greater similarity to the original questionnaire. It would be advisable in future studies to make an assessment of the performance of the instrument after removing the potentially redundant items.

No floor or ceiling effects were present in our study. This indicates our questionnaire seems able to distinguish patients with the lowest or highest possible score [28].

Regarding convergent validity, we found high positive intercorrelations between the Spanish-language version of the PGQ and the rest of the questionnaires.

Concerning factorial structure of the PGQ, the results of exploratory factor analysis identified a one-dimensional structure of the PGQ: the results of the matrix of components showed two factors, explained with over 61.11% of the variance. First factor explains 55.08% of the variance; the second factor explained 6.03% of the variance and the slope of the scree plot began to change in the second factor (Fig. 3). The presence of these two factors may explain the two subscales of symptoms and activity.

In the study of Stuge et al. [4] the factor structure of both subscales (activity and symptoms) are analysed separately and it is determined by a Rasch analysis that both are one-dimensional. In this study the two subscales are not analysed as a whole.

A more precise study of the factorial structure of the PGQ may be made by a confirmatory factor analysis thereof. This analysis requires a larger sample size (minimum 200 subjects) [33].

In contrast to the original questionnaire, the Spanish version of the PGQ seems not to have a discriminatory capacity between pregnant / postpartum women and 3 areas of pain / 1 or 2 areas of pain. A probable reason to explain this difference is only 6 participants in our sample reported pain in the 3 areas. This is possibly why the discriminant analysis between areas was not significant [29].

Test-retest reliability showed a very high level of consistency. This strong correlation indicates a high reliability of the Spanish version of the PGQ. Therefore, our tool provides stable and consistent results when repeated over time [28].

### Clinical implications

The PGQ is indicated in the assessment of PGP in pregnant and postpartum women.

Currently there is no instrument in Spanish that measures disability due to PGP, so creating this version of the PGQ is a new validation tool for specialists in this area. Validation thereof provides a specific tool for the assessment of PGP associated with pregnancy.

### Limitations

Patients were recruited using a series of clinical tests. These tests were provided to specialists as a reference for diagnosis. A limitation of our study is no statistical analysis of this data has been done. An analysis of the results of these tests and their correlation with the results of the questionnaire could have been allow a more accurate statistical analysis of the sample. We tried to compensate for the absence of these results including the initial verification questions, which in turn attempted to improve the representativeness of the sample.

Another limitation is this study did not assess the sensitivity to change of the instrument because the calculation of this psychometric feature requires a longitudinal study.

### Recommendations for future research

A larger sample, of at least 200 participants, is needed to confirm findings about unidimensionality of the instrument. It could also provide a larger number of subjects referring pain in 3 regions. Thus, the discriminatory capacity of the instrument depending on the number of areas of referred pain could be studied in a better way.

It would be also advisable for future studies to make an assessment of the performance of the instrument after removing the potentially redundant items.

Furthermore, performing a longitudinal study would be recommended to assess the sensitivity to change of the instrument.

## Conclusions

The Spanish-language version of the PGQ is a valid and reliable instrument for the assessment of PGP disability in pregnant and postpartum women. It is an understandable and user-friendly instrument that provides Spanish clinical practice with an assessment tool unavailable until now. It facilitates decision-making and research by establishing common international references.

The adaptation of this questionnaire encourages the research and development of clinical practice concerning PGP. The use of this new tool will facilitate international research by establishing common references. The use of PGQ is indicated in countries with Spanish speaking populations.

## Additional file

Additional file 1: Spanish version of the PGQ. (PDF 693 kb)

## Abbreviations

ICC: intraclass correlation coefficient
PGP: pelvic girdle pain
PGQ: pelvic girdle questionnaire
ROC: receiver operating characteristic
SEM: standard error of measurement

## References

[1] Elden H, Gutke A, Kjellby-Wendt G, Fagevik-Olsen M, Ostgaard H-C (2016). Predictors and consequences of long-term pregnancy-related pelvic girdle pain: a longitudinal follow-up study. BMC Musculoskelet Disord.

[2] Fagevik Olsén M, Elden H, Gutke A (2014). Evaluation of self-administered tests for pelvic girdle pain in pregnancy. BMC Musculoskelet Disord.

[3] Wu WH, Meijer OG, Uegaki K, Mens JMA, van Dieën JH, Wuisman PIJM (2004). Pregnancy-related pelvic girdle pain (PPP), I: Terminology, clinical presentation, and prevalence. Eur Spine J.

[4] Malmqvist S, Kjaermann I, Andersen K, Økland I, Larsen JP, Brønnick K (2015). The association between pelvic girdle pain and sick leave during pregnancy; a retrospective study of a Norwegian population. BMC Pregnancy Childbirth.

[5] Vleeming A, Albert HB, Ostgaard HC, Sturesson B, Stuge B (2008). European guidelines for the diagnosis and treatment of pelvic girdle pain. Eur Spine J.

[6] Ostgaard HC, Zetherström G, Roos-Hansson E, Svanberg B (1994). Reduction of back and posterior pelvic pain in pregnancy. Spine.

[7] Östgaard HC. Point of View: Pain Pattern in Pregnancy and “catching” of the Leg in Pregnant Women With Posterior Pelvic Pain. Spine [Internet]. 1997 Aug 15 [cited 2016 Sep 1];22(16). Available from: http://insights.ovid.com/spine/spne/1997/08/150/point-view-pain-pattern-pregnancy-catching-leg/14/00007632 10.1097/00007632-199708150-00013 9280024

[8] Albert H, Godskesen M, Westergaard J (2001). Prognosis in four syndromes of pregnancy-related pelvic pain. Acta Obstet Gynecol Scand.

[9] Stuge B, Garratt A, Krogstad Jenssen H, Grotle M (2011). The pelvic girdle questionnaire: a condition-specific instrument for assessing activity limitations and symptoms in people with pelvic girdle pain. Phys Ther.

[10] Grotle M, Garratt AM, Krogstad Jenssen H, Stuge B (2012). Reliability and construct validity of self-report questionnaires for patients with pelvic girdle pain. Phys Ther.

[11] Ostgaard HC, Zetherström G, Roos-Hansson E (1994). The posterior pelvic pain provocation test in pregnant women. Eur Spine J.

[12] Mens JM, Vleeming A, Snijders CJ, Koes BW, Stam HJ (2001). Reliability and validity of the active straight leg raise test in posterior pelvic pain since pregnancy. Spine.

[13] Vleeming A, de Vries HJ, Mens JMA, van Wingerden J-P (2002). Possible role of the long dorsal sacroiliac ligament in women with peripartum pelvic pain. Acta Obstet Gynecol Scand.

[14] Albert H, Godskesen M, Westergaard J (2000). Evaluation of clinical tests used in classification procedures in pregnancy-related pelvic joint pain. Eur Spine J.

[15] Fagevik Olsén M, Gutke A, Elden H, Nordenman C, Fabricius L, Gravesen M (2009). Self-administered tests as a screening procedure for pregnancy-related pelvic girdle pain. Eur Spine J.

[16] Beaton DE, Bombardier C, Guillemin F, Ferraz MB (2000). Guidelines for the process of cross-cultural adaptation of self-report measures. Spine.

[17] Pérez JE MA. Validez de contenido y juicio de expertos: una aproximación a su utilización. Av En Medición. 2008;6(1):27–36.

[18] Eysenbach G. Improving the Quality of Web Surveys: The Checklist for Reporting Results of Internet E-Surveys (CHERRIES). J Med Internet Res [Internet]. 2004 Sep 29 [cited 2016 Mar 7];6(3). Available from: http://www.ncbi.nlm.nih.gov/pmc/articles/PMC1550605/10.2196/jmir.6.3.e34 PMC1550605 15471760

[19] Alcántara-Bumbiedro S, Flórez-García MT, Echávarri-Pérez C, García-Pérez F (2006). Escala de incapacidad por dolor lumbar de Oswestry. Rehabilitación.

[20] Kovacs FM, Muriel A, Medina JM, Abraira V, Sánchez MDC, Jaúregui JO (2006). Psychometric characteristics of the Spanish version of the FAB questionnaire. Spine.

[21] García Campayo J, Rodero B, Alda M, Sobradiel N, Montero J, Moreno S (2008). Validation of the Spanish version of the Pain Catastrophizing Scale in fibromyalgia. Med Clínica.

[22] Vallès J, Guilera M, Briones Z, Gomar C, Canet J, Alonso J (2010). Validity of the Spanish 8-item short-form generic health-related quality-of-life questionnaire in surgical patients: a population-based study. Anesthesiology.

[23] Lundeberg T, Lund I, Dahlin L, Borg E, Gustafsson C, Sandin L (2001). Reliability and responsiveness of three different pain assessments. J Rehabil Med.

[24] Kline RB (2010). Principles and Practice of Structural Equation Modeling.

[25] Aiken LR (1980). Content Validity and Reliability of Single Items or Questionnaires. Educ Psychol Meas.

[26] Penfield RD, Peter R, Giacobbi J (2004). Applying a Score Confidence Interval to Aiken’s Item Content-Relevance Index. Meas Phys Educ Exerc Sci.

[27] Andresen EM (2000). Criteria for assessing the tools of disability outcomes research. Arch Phys Med Rehabil.

[28] Terwee CB, Bot SDM, de Boer MR, van der Windt DAWM, Knol DL, Dekker J (2007). Quality criteria were proposed for measurement properties of health status questionnaires. J Clin Epidemiol.

[29] Altman DG (1990). Practical Statistics for Medical Research.

[30] Mokkink LB, Terwee CB, Patrick DL, Alonso J, Stratford PW, Knol DL (2010). The COSMIN study reached international consensus on taxonomy, terminology, and definitions of measurement properties for health-related patient-reported outcomes. J Clin Epidemiol.

[31] Haley SM, Fragala-Pinkham MA (2006). Interpreting change scores of tests and measures used in physical therapy. Phys Ther.

[32] Nunnally JC, Bernstein H. Psychometric theory. New York: McGraw-Hill; 1978. p. 730.

[33] Manuel Batista-Foguet J, Coenders G, Alonso J (2004). Análisis factorial confirmatorio. Su utilidad en la validación de cuestionarios relacionados con la salud. Med Clínica.

