# Supplementary material for: Transcultural adaptation and psychometric validation of a Spanish-language version of the “Pelvic Girdle Questionnaire”
Source: Health Qual Life Outcomes. 2017 Feb 1;15:30. doi: 10.1186/s12955-017-0605-0 (PMC5286739; doi:10.1186/s12955-017-0605-0)
Supplement: Additional file 1: — Spanish version of the PGQ. (PDF 693 kb) [file 12955_2017_605_MOESM1_ESM.pdf]

APPENDIX
Cuestionario de la cintura pélvica

Nombre:________Fecha:____________

¿En qué medida su dolor a nivel de la pelvis dificulta la realización de las actividades citadas a continuación? Para cada actividad, marque la casilla que mejor describa cómo se siente hoy.

**Indique en qué medida le afecta el dolor de la pelvis a la hora de realizar las siguientes actividades:**	**Nada (0)**	**Levemente(1)**	**Bastante(2)**	**Mucho (3)**
1. Vestirse				
2. Permanecer de pie más de 10 minutos				
3. Permanecer de pie más de 60 minutos				
4. Agacharse				
5. Estar sentado menos de 10 minutos				
6. Estar sentado más de 60 minutos				
7. Caminar menos de 10 minutos				
8. Caminar más de 60 minutos				
9. Subir las escaleras				
10. Realizar tareas domésticas				
11. Cargar objetos ligeros				
12. Cargar objetos pesados				
13. Levantarse/ sentarse				
14. Empujar un carrito de la compra				
15. Correr				
16. Llevar a cabo actividades deportivas*				
17. Tumbarse				
18. Darse la vuelta en la cama				
19. Llevar una vida sexual normal *				
20. Empujar algo con el pie				

*** Si alguna de estas actividades no procede por favor, marque la casilla de la derecha**

**Cuánto dolor siente :**	**Nada (0)**	**Un poco (1)**	**Bastante (2)**	**Mucho (3)**
21. Por la mañana				
22. Por la noche				

**En qué medida el dolor de la pelvis hace que:**	**Nada (0)**	**Levemente (1)**	**Bastante (3)**	**Mucho (3)**
23. Su pierna/ sus piernas ceda(n)				
24. Haga las cosas más lentamente				
25. Su sueño se vea interrumpido				
